# Evaluation of Helping Babies Breathe Quality Improvement Cycle (HBB-QIC) on retention of neonatal resuscitation skills six months after training in Nepal

**DOI:** 10.1186/s12887-017-0853-5

**Published:** 2017-04-11

**Authors:** Ashish KC, Johan Wrammert, Viktoria Nelin, Robert B. Clark, Uwe Ewald, Stefan Peterson, Mats Målqvist

**Affiliations:** 1 International Maternal and Child Health, Department of Women’s and Children’s Health, Uppsala University, Uppsala, Sweden; 2 United Nation’s Children’s Fund (UNICEF), Nepal Country Officer, UN House, Lalitpur, Nepal; 3 Latter-day Saint Charities, Salt Lake City, UT USA; 4 Global Health, Public Health Services, Karolinska Institute, Stockholm, Sweden; 5 School of Public Health, Makerere University, Kamala, Uganda; 6 Health Section, Programme Division, United Nations Children Fund (UNICEF), United Nations Plaza, New York, NY 10017 USA

**Keywords:** Neonatal resuscitation, Helping babies breathe, Retention of skills, Multi-faceted implementation strategy, Quality improvement cycle, Nepal

## Abstract

**Background:**

Each year 700,000 infants die due to intrapartum-related complications. Implementation of Helping Babies Breathe (HBB)-a simplified neonatal resuscitation protocol in low-resource clinical settings has shown to reduce intrapartum stillbirths and first-day neonatal mortality. However, there is a lack of evidence on the effect of different HBB implementation strategies to improve and sustain the clinical competency of health workers on bag-and-mask ventilation. This study was conducted to evaluate the impact of multi-faceted implementation strategy for HBB, as a quality improvement cycle (HBB-QIC), on the retention of neonatal resuscitation skills in a tertiary hospital of Nepal.

**Methods:**

A time-series design was applied. The multi-faceted intervention for HBB-QIC included training, daily bag-and-mask skill checks, preparation for resuscitation before every birth, self-evaluation and peer review on neonatal resuscitation skills, and weekly review meetings. Knowledge and skills were assessed through questionnaires, skill checklists, and Objective Structured Clinical Examinations (OSCE) before implementation of the HBB-QIC, immediately after HBB training, and again at 6 months. Means were compared using paired t-tests, and associations between skill retention and HBB-QIC components were analyzed using logistic regression analysis.

**Results:**

One hundred thirty seven health workers were enrolled in the study. Knowledge scores were higher immediately following the HBB training, 16.4 ± 1.4 compared to 12.8 ± 1.6 before (out of 17), and the knowledge was retained 6 months after the training (16.5 ± 1.1). Bag-and-mask skills improved immediately after the training and were retained 6 months after the training. The retention of bag-and-mask skills was associated with daily bag-and-mask skill checks, preparation for resuscitation before every birth, use of a self-evaluation checklist, and attendance at weekly review meetings. The implementation strategies with the highest association to skill retention were daily bag-and-mask skill checks (RR-5.1, 95% CI 1.9–13.5) and use of self-evaluation checklists after every delivery (RR-3.8, 95% CI 1.4–9.7).

**Conclusions:**

Health workers who practiced bag-and-mask skills, prepared for resuscitation before every birth, used self-evaluation checklists, and attended weekly review meetings were more likely to retain their neonatal resuscitation skills. Further studies are required to evaluate HBB-QIC in primary care settings, where the number of deliveries is gradually increasing.

**Trial registration:**

ISRCTN97846009. Date of Registration- 15 August 2012.

## Background

Globally, 2.9 million neonates die every year, of which 700,000 die due to intrapartum-related complications [1]. The first minute after a baby is born—the “Golden minute™”—is the crucial window for the initiation of neonatal resuscitation among the 10 million non-breathing babies born annually [2]. Neonatal resuscitation competency among health workers is therefore critical in every delivery room to ensure the safety and health of newborn infants. Resuscitation training in facilities has been shown to reduce the number of intrapartum-related neonatal deaths by 30% [3]. Although health workers are the key personnel in the provision of neonatal resuscitation, it has been revealed that in low-resource settings staff usually have inadequate training and knowledge, and that additional formal guidance and support are needed [4, 5].

Nepal has a neonatal mortality rate of 23 per thousand live births and intrapartum-related complications account for 23% of neonatal deaths [1, 6]. In Nepal, more than half of all deliveries take place at health institutions, but the quality of resuscitation provided at the time of birth has been found to be inadequate [7]. Identifying the implementation strategies that could improve and sustain neonatal resuscitation competency will be key to improve the quality of resuscitation care provided.

There are a number of intervention strategies that have been identified to improve the performance and competencies of health workers for a number of other clinical guidelines [8]. However, strategies to improve and sustain competency on neonatal resuscitation have not been evaluated.

Helping Babies Breathe® (HBB), developed by the American Academy of Pediatrics, is designed to train birth attendants in developing countries on the essential skills of neonatal resuscitation [9]. It is based on evidence from a neonatal evaluation study done by ILCOR (International Liaison Committee on Resuscitation), and recognizes that in many countries only one birth attendant is present to provide care to both the mother and the newborn [10, 11].

The educational material of HBB consists of a well-tested pictorial representation of the resuscitation protocol, learner workbooks, facilitator flip charts, neonatal simulators and the required equipment – i.e., reusable ventilation bag-and-mask devices and bulb suction devices [9]. An evaluation of the HBB program in Tanzania showed that intrapartum-related stillbirth and early neonatal mortality was reduced by 24% and 47%, respectively [12]. The Tanzanian study showed that the HBB program is a low-cost intervention, with the estimated cost per life saved at USD 233 and USD 4.21 per life year gained [13]. However, implementation strategies for HBB in the Tanzania study were not evaluated in relation to the retention of neonatal resuscitation competencies among health workers.

An evaluation study conducted in Ethiopia found that HBB training improved the neonatal resuscitation knowledge of health workers immediately after training and also eliminated the knowledge difference that was present prior to training among health workers [14]. A study from Rwanda revealed that neonatal resuscitation competency dropped to an unsatisfactory level 3 months after the training, indicating that training alone was not adequate to retain the health worker’s neonatal resuscitation knowledge and skills [15]. The proportion of providers at a rural hospital in Tanzania who were competent in simulated routine care and neonatal resuscitation scenarios increased after HBB training, and their knowledge remained at 7 months after training; however, the improvement did not transfer into clinical competency [16]. There is thus a need to further evaluate multi-faceted interventions to promote retention of resuscitation skills, as well as to make sure that knowledge is translated into clinical practice.

We therefore conducted this study to evaluate the impact of a multi-faceted implementation strategy for HBB as a Quality Improvement Cycle (HBB-QIC), on change and retention of health workers’ knowledge and skills of neonatal resuscitation.

## Methods

We conducted this study in a tertiary hospital in Kathmandu, Nepal. In 2011, the hospital had around 22,000 deliveries, and an early neonatal mortality of nine per thousand live births [17]. The hospital provides level III obstetric and gynecological services and is publicly funded.

Labor and delivery services are provided in the admission unit, antenatal care unit, labor unit, maternal and newborn service center (MNSC), and the operation theatre. The hospital also has a system that rotates the staff of these units on a periodic basis.

This was a sub-set of a larger study conducted to evaluate the change in perinatal mortality before and after the implementation of a simplified neonatal resuscitation protocol, the HBB-QIC, in the hospital [18]. The duration of the larger study was 18 months, from July 2012 to September 2013. As part of the larger study evaluating the impact of neonatal resuscitation protocol implementation, the study received approval from the Hospital’s Institutional Review Committee, the Nepal Health Research Council (Reg. No. 37/2012) and the Ethical Review Board of Uppsala University (dnr 2012/267). The study was registered as clinical trial, ISRCTN 97846009 [18].

All 137 nurses working in the admission unit, antenatal care unit, labor unit, MNSC, operation theatre and postnatal ward were included in the study. Informed written consent was obtained from each health worker participating in the study.

### Study design

This was a time-series design where a series of periodic measurements were completed to evaluate our multi-faceted implementation strategy for the HBB-QIC, at three different time points (Table 1).

**Table 1:** Evaluation of HBB QIC

	Thematic area	Tools	Evaluation design
Knowledge and skill competency	Knowledge assessment	17 multiple choice questions	Before training, immediately after training and 6 months after training
	Bag-and-mask skill	7 step skill observation checklist	Before training, immediately after training and 6 months after training
	Preparation at birth	5 step skill observation checklist	Before training, immediately after training and 6 months after training
	OSCE A	13 step observation checklist	Before training, immediately after training and 6 months after training
	OSCE B	18 step observation checklist	Before training, immediately after training and 6 months after training
Clinical practice	Daily skill check	7 step skill observation checklist	Observation on a daily basis using a checklist
	Preparation for resuscitation before every birth	5 step skill observation checklist	Observation on a daily basis using a checklist
	Self-evaluation checklist	21 step checklist	Observation on a daily basis using a checklist
	Peer evaluation process	HBB schematic protocol	Observation on a daily basis using a checklist
	Weekly review meetings	Notes of the meeting	Observation on a daily basis using a checklist

The knowledge and skills of health workers were assessed using the standard tools included in the HBB package, which were validated and used in other settings where HBB knowledge and skill evaluations have been conducted [9]. A questionnaire with 17 multiple-choice questions was used to assess the knowledge of health workers on the HBB protocol. The following tools were used to evaluate the skills of health workers in using the HBB protocol: a 7-step checklist for bag-and-mask skill checks, a 5-step checklist for preparation for resuscitation before every birth skill checks, a 13-step checklist with simulation for the first Objective Structured Clinical Examination (OSCE A), and a 18-step checklist with simulation for OSCE B. Two OSCEs tested healthcare worker’s ability to prepare for, assess, and act in a scenario of routine newborn care (OSCE A) and a scenario requiring bag-and-mask ventilation (OSCE B) [9]. Successful completion of each OSCE required correct overall performance (≥80%), as well as the completion of key assessments and interventions such as “recognizes baby not breathing/crying” and “provides bag-and-mask ventilation” [9]. Assessment using these tools was completed before the HBB training was provided, immediately after the training, and again at 6 months after completion of the training (Table 1).

Information on each health worker’s daily routines, including daily bag-and-mask skill checks, preparation for resuscitation before every birth, self-evaluation checklist usage, completion of the peer evaluation process, and attendance at the weekly review meetings was assessed using a direct observation checklist (Table 1).

### HBB QIC intervention

The HBB-QIC was implemented from January until September 2013. The HBB-QIC included multi-faceted implementation strategy including: HBB training, daily bag-and-mask skill checks, preparation for resuscitation before every birth, self-evaluation and peer review on neonatal resuscitation skills and weekly review meetings (Table 2).

**Table 2:** Description of the multi-faceted implementation strategy for Helping Babies Breathe (HBB) Quality Improvement Cycle (QIC)

Component	Activity	Facilitators and participants
HBB training	Two-day training: First day on HBB knowledge and skills as per standard package and second day on components of HBB QIC standards, training of trainers on how to conduct weekly review meeting, how to fill self-evaluation checklists and conduct peer evaluations.	Facilitators: HBB trainers Participants: Staff of the delivery units
Setting up HBB QIC standards	At each unit: Development of QIC goals and objectives, development of a place for daily bag-and-mask skill checks, QIC weekly review meetings, use of self-evaluation checklists and peer reviews after each resuscitation.	Facilitators: Study team Participants: Staff of the delivery units
QIC Weekly review meeting	At each unit, the unit in-charge facilitates the weekly review meetings on the progress of implementation of HBB QIC standards.	Facilitators: HBB trainers Participants: Staff of the delivery units
Daily bag-and-mask skill check	At each unit, each staff does a bag-and-mask skill check on a mannequin before starting duty.	Facilitators: Unit in-charge Participants: Staff of the delivery units
Self-evaluation checklist after each delivery	A self-evaluation checklist, which consists of a list of steps for immediate newborn care and neonatal resuscitation as per HBB protocol with checkboxes. After completing care of each newborn, the nurse midwife will fill up the self-evaluation checklist based on the steps completed as per the HBB protocol.	Facilitators: Unit in-charge Participants: Staff of the delivery units
Peer review after each resuscitation	A mounted poster with the steps of the HBB protocol will be attached at each resuscitation table, so that peers can review with the colleague completing resuscitation on whether the steps were followed.	Facilitators: Unit in-charge Participants: Staff of the delivery units

First, a two-day training package on the HBB-QIC was provided to all hospital staff working in the three delivery units. There were 6 trainers who trained all the staff in each of the delivery units in a cascade manner. During the study period, there was no rotation of staff among the delivery units. Following the training, bag-and-mask kits and penguin (bulb) suctions™ were provided to each delivery unit. Additionally, HBB mannequins were placed at the entry of each delivery unit for daily skill checks; self-evaluation checklists were also attached to each clinical record form; and HBB schematic posters were placed in front of each resuscitation table for peer review. Weekly review meetings were conducted by each unit in-charge to discuss progress on the implementation of HBB-QIC standards.

### Data management

In order to complete sampling and data collection, an independent surveillance team was formed. The team consisted of eight female surveillance officers with an academic background in nurse-midwifery and sufficient experience in clinical research. A research manager supervised the activities of this surveillance team. The surveillance team was trained to collect information using an observation checklist. Surveillance officers who conducted the HBB trainings collected information from the study participants, including demographic characteristics, academic qualifications and clinical experience.

After the surveillance officers collected this information, it was given to the data entry officer for dataset creation and management. The information was reviewed for completeness and then entered into an electronic database that was developed in the Census and Survey Processing System (CS Pro) software (US Census Bureau, ICF Macro). The data was then cleaned and transferred into the Statistical Package for the Social Sciences (SPSS) software (IBM Corporation) for data analysis.

### Data analysis

The background characteristics of the health workers that were analyzed included age, professional experience, number of deliveries attended per month, number of neonatal resuscitations conducted per month and academic qualifications. The mean ± SD and median (IQR) for age of the health worker were calculated; mean and median years of professional experience in midwifery were calculated; the mean and median number of deliveries and resuscitations attended per month were also calculated. Academic qualifications were categorized into two groups, those who had completed an intermediate level of education in nursing or who were axillary nurse midwives compared to those who had completed a bachelor in nursing or higher.

The mean numeric scores obtained on the knowledge questionnaire, preparation for resuscitation before every birth checklist, bag-and-mask skill check, and OSCEs A and B at baseline, immediately after the training, and at 6 months after the training were compared using paired t-tests. The proportion of health workers who scored ≥80% on the bag-and-mask skill checks before the training, immediately after the training, and at 6 months after the training was compared using paired t-tests.

The retention of bag-and-mask skills was calculated based on the change in bag-and-mask skill scores immediately after the training compared to 6 months after the training. The health workers who scored the same or better at 6 months after the training, as compared to immediately after training, were categorized as having retained their skills.

We then analyzed the association (*p* < 0.01) between retention of skills and health worker’s completion of HBB-QIC components – i.e., daily bag-and-mask skill checks, preparation for resuscitation before every birth, use of self-evaluation checklists, peer evaluation following each resuscitation occurrence, attendance at weekly meetings – as well as with the completion of at least one neonatal resuscitation per month using the Fischer’s exact test.

We also conducted logistic regression analysis to assess the level of association between the completion of each implementation strategy included in the HBB-QIC and the retention of bag-and-mask skills at 6 months.

Missing data that occurred randomly was adjusted for using the multiple imputation method [19].

## Results

All 137 health workers included in this study participated in knowledge and skill evaluation before the HBB training, immediately after training and at 6 months after the training. The mean age of the health workers was 31.8 ± 10.2 years and the median age was 27.0 (IQR 24.0–39.0) years. Among the health workers, the mean number of professional years of experience was 10.5 ± 3.3 years and the median was 9 (IQR 6.0–15.0) years. The mean number of deliveries attended by each health worker was 8.0 ± 5.5 and the median was 7.0 (5.0–9.0). The mean number of resuscitations conducted by each health worker per month was 3.0 ± 1.5 and median was 3.0 (2.0–4.0). More than half, 65%, of health workers had intermediate level nursing qualifications or were axillary nurse-midwives, and the remaining health workers had bachelor degrees in nursing or higher (Table 3).

**Table 3:** Background characteristics of the Health workers-nurse

Background Characteristics	Mean ± SD	Median (IQR)
Age in complete years	31.8 ± 10.2	27.0 (24.0–39.0)
Professional experience in midwifery in complete years	10.5 ± 3.3	9.0 (6.0–15.0)
Number of deliveries attended per month	8.0 ± 5.5	7.0 (5.0–9.0)
Number of resuscitation	3.0 ± 1.5	3.0 (2.0–4.0)
Academic Qualifications	Frequency *N* = 137 (%)	
Axillary nurse midwives/intermediate in nursing	90 (65.7)	
Bachelor in nursing	47 (34.3)	

There was a significant change (<0.001) in the mean knowledge score after the HBB training, 16.4 ± 1.4 compared to 12.8 ± 1.6, and the knowledge was retained 6 months after the training (16.5 ± 1.1) (*p* = 0.6). There was a significant change (<0.001) from baseline in the mean skills scores for preparation for resuscitation before every birth, bag-and-mask skills, OSCE A, and OSCE B after the training, which was retained 6 months after the training (Table 4).

**Table 4:** Changes in knowledge and completion of preparation at birth, bag-and-mask skill checks, and OSCEs before, immediately after and 6 months after HBB training

	Before the training (*N* = 137)	Immediately after training (*N* = 137)		Follow up after 6 months (*N* = 137)	
	Mean ± SD	Mean ± SD	*p*-value	Mean ± SD	*p*-value
Knowledge (out of 17)	12.8 ± 1.6	16.4 ± 1.4	<0.001	16.5 ± 1.1	0.6
Preparation for resuscitation before every birth (out of 5)	2.2 ± 0.5	4.7 ± 0.1	<0.001	4.8 ± 0.03	0.1
Bag and Mask (out of 7)	2.3 ± 0.5	6.7 ± 0.6	<0.001	6.7 ± 0.5	0.815
OSCE A (out of 13)	3.9 ± 2.6	12.3 ± 0.9	<0.001	12.2 ± 0.8	0.07
OSCE B (out of 18)	3.7 ± 1.6	16.5 ± 1.2	<0.001	16.8 ± 1.1	*p* = 0.1

**Fig. 1:**
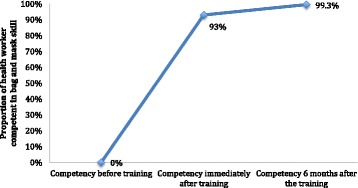
Change in the proportion of the health worker who were competent (≥80%) in bag and mask skill

There were no health workers who were competent in bag-and-mask skills before the training; 93% of health workers were competent immediately after the training (*p* < 0.001); and 99% of the health workers were competent 6 months after the training (*p* = 0.8) (Fig. 1).

More than half of the health workers practiced their bag-and-mask skills daily, 61% of them prepared for resuscitation before every birth, 82% of them used self-evaluation checklists after every birth, 43% of them completed peer evaluation after each resuscitation and 85% of them attended weekly review meetings (Table 5).

**Table 5:** Level of participation in the Helping Babies Breathe (HBB) Quality Improvement Cycle (QIC)

	Frequency (*N* = 137)	Percent
Frequency of bag-and-mask skill checks		
Every day	71	51.8
2 times a week	46	33.6
Weekly	20	14.6
Frequency of preparation for resuscitation before every birth		
At every birth	84	61.3
Occasionally	30	38.7
Frequency of practicing self-evaluation		
For every birth	112	81.8
Occasionally	25	18.2
Frequency of practicing peer evaluation		
For every resuscitation	59	43.0
Occasionally	78	57.0
Attendance of weekly review meeting		
Yes	117	85.4
No	20	14.6

There was a positive association (*p* < 0.01) between retention of bag-and-mask skills and health worker’s completion of daily bag-and-mask skill checks, preparation for resuscitation before every birth, use of self-evaluation checklists and attendance at weekly review meetings (Table 6).

**Table 6:** Association between the different implementation strategies of the Helping Babies Breathe (HBB) Quality Improvement Cycle (QIC) and the retention of bag-and-mask skills

		Non-retention (*N* = 27)	Retention (*N* = 110)	*p*-value
Daily bag-and-mask skill checks	Yes	6 (22.2%)	65(40.9%)	*p* = 0.001
	No	21 (77.8%)	45 (59.1%)	
Preparation for resuscitation before every birth	Yes	12(44.4%)	72(65.5%)	*p* = 0.038
	No	15 (55.6%)	38 (34.5%)	
Use of self-evaluation checklist	Yes	17 (63.0%)	95(86.4%)	*p* = 0.008
	No	10 (37.0%)	15 (13.6%)	
Peer evaluation after each resuscitation	Yes	12 (44.4%)	47 (42.7%)	*p* = 0.5
	No	15 (55.6%)	63 (57.3%)	
Attendance of weekly review meetings	Yes	20 (74.1%)	97 (88.2%)	*p* = 0.065
	No	7 (25.9%)	13 (11.8%)	

The regression analysis showed that health workers who conducted daily bag-and-mask skill checks were five times more likely to retain neonatal resuscitation skills than those who did not (RR-5.1, 95% CI 1.9–13.5). Health workers who prepared for resuscitation before every birth had a two-fold increased likelihood of retaining the skills than those who did not (RR-2.4, 95% CI 1.0–5.6). Health workers who used self-evaluation checklists after each delivery were four times more likely to retain their skills than those who did not (RR-3.8, 95% CI 1.4–9.7). Health workers that attended weekly meetings had a 2.6 times increased likelihood of retaining their skills than those who did not attend (RR-2.6, 95% CI 1.0–7.4) (Table 7).

**Table 7:** Logistic regression analysis on the level of association between different implementation strategies of the Helping Babies Breathe (HBB) Quality Improvement Cycle (QIC) and bag-and-mask skill retention

HBB QIC	Relative Risk	95% CI
Bag and mask skill check daily	5.1	1.9–13.5
Preparation for resuscitation before every birth	2.4	1.0–5.6
Use of self-evaluation checklist	3.8	1.4–9.7
Attendance of weekly review meeting	2.6	1.0–7.4

## Discussion

We applied different implementation strategies as part of a HBB-QIC to improve the knowledge and competency at different time points during the intervention period. Our study showed health workers who conducted bag-and-mask skill checks daily, who were prepared for resuscitation before every birth, who used self-evaluation checklists after each delivery, and those who attended weekly review meetings were more likely to retain their neonatal resuscitation skills, even at 6 months after the completion of training.

There are, however, certain limitations to our study. First, this study had a time-series design, thus the effectiveness of each individual component within the HBB-QIC cannot be evaluated, and we can only derive the association between the implementation strategy as a whole and the retention of neonatal resuscitation skills. Second, the use of self-evaluation checklists was evaluated based on whether these forms were completed correctly by the participants along with the other forms that had to be completed following every delivery. Therefore, there may be some discrepancy between actual practice and what was recorded. The final limitation was the presence of surveillance officers to observe the implementation of the HBB QIC, especially the bag-and-mask skill checks and weekly review meetings, which might have a Hawthorne effect (observer’s bias) in changing health worker’s behaviors. The strength of our study was that we used a tested tool to evaluate the neonatal resuscitation knowledge and skills of the health worker [9].

Several studies in high-income and low-income countries have shown that resuscitation knowledge and skills improve immediately following training, however, these resuscitation skills tend to deteriorate over a period of time [15, 20–25]. Therefore, neonatal resuscitation training in itself is not an effective implementation strategy to retain resuscitation skills. Similar to our findings from this study, a study done in Canada has shown that the review of schematic posters on neonatal resuscitation before or after resuscitation of babies is not an effective strategy for retention of neonatal resuscitation skills [26].

Several systematic reviews have shown that the review of clinical performance among health workers on a periodic basis provides an opportunity to share the challenges faced in the clinical setting and to improve the clinical skills and performance as a team, especially if the baseline performance on the clinical protocol is poor [27–30]. We showed that weekly review meetings among the nursing staff on the clinical performance of resuscitation helped to improve resuscitation skills to a moderate extent, as the baseline neonatal resuscitation competency was poor in this setting. Systematic reviews have also shown that a combination of implementation strategies (such as weekly review meetings, periodic simulated skill checks, checklists, and self-evaluation) is a more effective strategy to improve clinical performance as compared to the use of a singular strategy [31–36]. However, these implementation strategies were never implemented in combination to improve and increase the retention of neonatal resuscitation skills.

In the context of Nepal, the current number of neonatal deaths due to intrapartum-related complications needs to be reduced by at least three quarters in order to reach the pledged Every Newborn Action Plan target of a neonatal mortality of 10 or less per 1000 live birth by 2035 [37]. Further, the quality of neonatal resuscitation needs to be improved from that of the current state by improving health worker performance of neonatal resuscitation [7, 38]. Health workers need to be fully competent in neonatal resuscitation skills, and further need to be able to retain this competency such that knowledge is translated into clinical practice to reduce the burden of intrapartum-related death and morbidity. Nepal’s current national strategy to provide skilled attendance at birth through neonatal resuscitation training programs is not adequate to sustain clinical competency of neonatal resuscitation as shown by several previous studies [38–41]. A combination of strategies should be considered for implementation in health facilities across the country.

## Conclusion

To our knowledge this is the first study done to evaluate the impact of a multi-faceted implementation strategy of HBB on the retention of neonatal resuscitation skills in low-income settings. We demonstrate that health workers, who conduct bag-and-mask skill checks daily, make preparation for resuscitation at every birth, use self-evaluation checklists after each delivery, and attend weekly review meetings have a higher likelihood for retention of neonatal resuscitation skills, even at 6 months post-training. Since, the HBB-QIC was implemented in a setting with a high number of deliveries, with a large team of nursing staff in each delivery unit, the implementation strategy such as weekly review meetings can be suitable in similar settings, but may not be suitable in primary care settings where there are fewer nursing staff. The other effective implementation strategies of daily bag-and-mask skill checks, preparation for resuscitation at every birth, and use of self-evaluation checklists can be applied in primary care settings to improve retention of resuscitation skills. However, the combination of different strategies needs to be evaluated in primary care settings, the point of access for delivery services among most women in rural areas. The innovative aspect of HBB-QIC is the multi-faceted implementation strategy approach. Future research should focus on further determining and refining those components that improve retention of neonatal resuscitation skills and clinical performance.

## Abbreviations

HBB: Helping Babies Breathe
QIC: Quality Improvement Cycle
MNSC: Maternal Newborn Service Center
OSCE: Objective structured clinical evaluation
IQR: Inter quartile range
SD: Standard deviation
RR: Relative risk
